# Comparative genomics and transcriptomics of trait-gene association

**DOI:** 10.1186/1471-2164-13-669

**Published:** 2012-11-26

**Authors:** Sebastián Aguilar Pierlé, Michael J Dark, Dani Dahmen, Guy H Palmer, Kelly A Brayton

**Affiliations:** 1 Program in Genomics, Department of Veterinary Microbiology and Pathology, Paul G. Allen School for Global Animal Health, Washington State University, Pullman, WA, 99164-7040, USA; 2 Department of Infectious Diseases and Pathobiology, College of Veterinary Medicine, University of Florida, Gainesville, FL, 32611-0880, USA; 3 Emerging Pathogens Institute, University of Florida, Gainesville, FL, 32611-0880, USA

**Keywords:** Bacteria, Rickettsia, SNP, RNA-seq, Anaplasma

## Abstract

**Background:**

The Order *Rickettsiales* includes important tick-borne pathogens, from *Rickettsia rickettsii*, which causes Rocky Mountain spotted fever, to *Anaplasma marginale*, the most prevalent vector-borne pathogen of cattle. Although most pathogens in this Order are transmitted by arthropod vectors, little is known about the microbial determinants of transmission. *A. marginale* provides unique tools for studying the determinants of transmission, with multiple strain sequences available that display distinct and reproducible transmission phenotypes. The closed core *A. marginale* genome suggests that any phenotypic differences are due to single nucleotide polymorphisms (SNPs). We combined DNA/RNA comparative genomic approaches using strains with different tick transmission phenotypes and identified genes that segregate with transmissibility.

**Results:**

Comparison of seven strains with different transmission phenotypes generated a list of SNPs affecting 18 genes and nine promoters. Transcriptional analysis found two candidate genes downstream from promoter SNPs that were differentially transcribed. To corroborate the comparative genomics approach we used three RNA-seq platforms to analyze the transcriptomes from two *A. marginale* strains with different transmission phenotypes. RNA-seq analysis confirmed the comparative genomics data and found 10 additional genes whose transcription between strains with distinct transmission efficiencies was significantly different. Six regions of the genome that contained no annotation were found to be transcriptionally active, and two of these newly identified transcripts were differentially transcribed.

**Conclusions:**

This approach identified 30 genes and two novel transcripts potentially involved in tick transmission. We describe the transcriptome of an obligate intracellular bacterium in depth, while employing massive parallel sequencing to dissect an important trait in bacterial pathogenesis.

## Background

The ongoing revolution in genome sequencing has enabled ever-increasing sequence generation at an ever-decreasing cost. The growing availability of fully sequenced genomes offers new opportunities to identify relationships between genotype and phenotype, one of the major goals of the genomics era. Comparative genomics were first introduced as a tool to predict trait-gene associations in 1998 while trying to define species-specific features of *Helicobacter pylori*[1]. This approach has been used to predict genomic determinants for well-known phenotypes, including hyperthermophily, flagellar motility and pili assembly [2-4]. These studies share the principle that species with similar phenotypes are likely to utilize orthologous genes in the involved biological process. Thus, the simultaneous presence of genes across species would suggest functional similarity among encoded proteins [5,6]. While these studies illustrate the advantages and applicability of this principle, they are dependent on previous knowledge of the genetic determinants of a specific trait.

The challenge of associating genes with phenotypes has been highlighted by the development of the pangenome concept and the abundance of intraspecies diversity that has been revealed. The pangenome of a bacterial species encompasses the sum of the genetic repertoire found in all strains [7]. Thus, it consists of the core genome found in all the strains plus the “accessory” genes unique to the different strains. Those bacterial species with a high number of accessory genes are termed “open” pangenomes, whereas those lacking strain specific genes are identified as “closed” pangenomes. While the “openness” of the pangenome is an obvious marker of diversity, sequence heterogeneity within the core gene set has also been shown to be relevant to natural genetic variation [8-10]. When several strains of *Streptococcus agalactiae* were compared to the 2603 VR strain, 99.2% of the total detected single nucleotide polymorphisms (SNPs) were unique to one strain, while none were common to all strains. A similar scenario was found between three strains of *Bacillus anthracis*, where all SNPs were unique to one strain. As these two organisms, are classical examples of open and closed pangenomes, respectively, this suggests that the SNP profile of a bacterial species can be open regardless of how “locked” their cores are.

An example of an organism with a closed core genome and a high degree of interstrain diversity is *Anaplasma marginale*, an obligate intracellular pathogen of both domestic and wild ruminants, with a small genome of 1.2 Mb for which the sequence of multiple strains has been determined [8,11,12]. No strain-specific genes and no plasmids were found among *sensu stricto* strains after sequencing of five strains [8,11]. In contrast, a high degree of allelic diversity was detected: global comparison of five strains revealed a total of 20,082 sites with SNPs detected in at least one of the analyzed strains and, with approximately 6,000 sites between any given pair. The high degree of gene content conservation suggests that phenotypic differences observed in *A. marginale* must be due to small polymorphisms between strains rather than whole gene insertions or deletions. Therefore, we exploited the interstrain diversity of *A. marginale* to map the genetic basis underlying phenotypic differences among strains.

*A. marginale* genome sequences are available for strains that clearly differ in a measurable phenotype: transmission by the arthropod vector. The Saint Maries, Puerto Rico, Virginia, EMø, 6DE and South Idaho strains are examples of efficiently transmitted strains [13-18]. The Florida strain, has been shown to have a very low transmission efficiency as it was not transmitted using >10 times the number of *Dermacentor andersoni* ticks routinely used for transmission with the St. Maries strain [17,19,20].

Due to the complete gene content conservation, differences in transmission efficiency in *A. marginale* are likely to be ascribed to sequence variation producing variant proteins or affecting gene transcription. Indeed, precedence is seen in bacterial pathogens, where SNPs have been discovered that provide a selective advantage in host colonization [21]. We combined two genomic sequencing approaches in order to find SNPs and transcriptional changes that segregate with transmission phenotype. We first compared the genome sequences of two strains, St. Maries and Florida, which display contrasting phenotypes with respect to the trait of interest, tick transmissibility. Candidate SNPs included polymorphisms encoding non-synonymous substitutions within genes, as well as SNPs located within putative promoter regions. Each SNP on the resulting list was evaluated through comparative genomics in three efficiently transmissible strains for its consistent segregation with phenotype. The remaining differences were sequenced in two additional efficiently transmissible strains. Only SNPs that were unique to the poorly transmissible Florida strain when compared to six efficiently transmitted strains were retained as candidates. This resulted in a list of candidate genes, consisting of those containing candidate SNPs or located downstream of putative promoter SNPs. Transcriptional analysis of candidate genes by RT-PCR revealed genes that were differentially transcribed in strains with distinctly different transmission efficiencies. To find additional transcriptional changes related to the phenotype of interest, we performed a genome wide transcriptome comparison using RNA-seq technology. Total mRNA populations from two *A. marginale* strains with different transmission capabilities were sequenced using three different platforms. This study makes use of two sequencing approaches and four different technologies to identify genes involved in a relevant microbial trait. We present, to our knowledge, the deepest analysis of an obligate intracellular bacterial transcriptome during the pathogen’s natural course of infection.

## Results

### Comparative genomics identifies SNPs that segregate with transmission status

**Figure 1:**
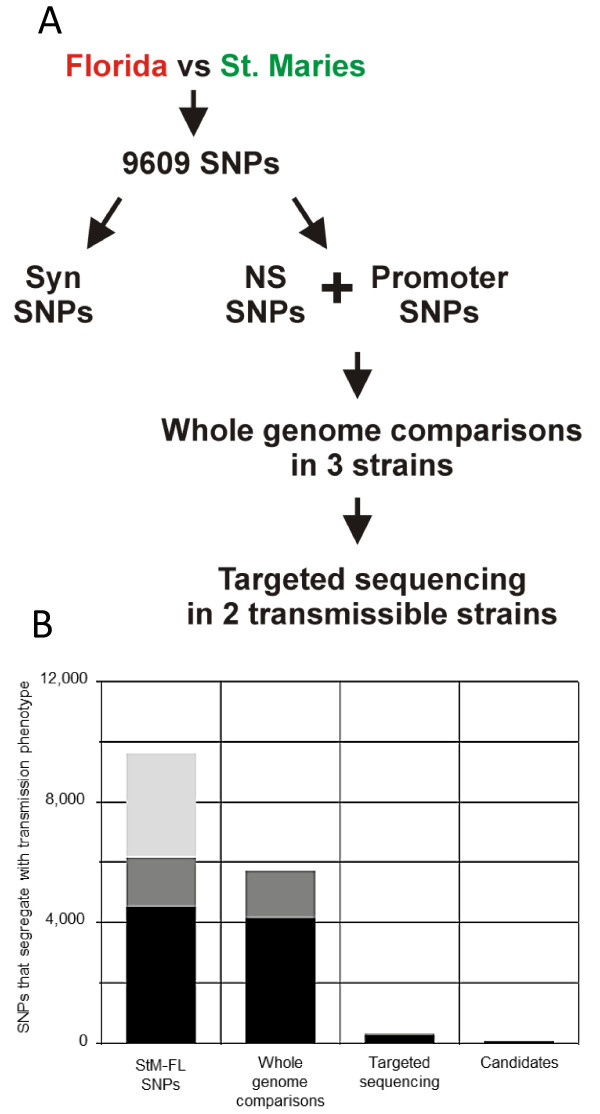
**SNPs segregated with transmission status through whole genome comparison and targeted sequencing. A**. Genome wide comparison of the non-transmissible Florida strain (red) with the efficiently transmitted St. Maries (green) strain produced 9609 SNPs. From this list we subtracted SNPs that encode for synonymous changes, leaving two types of SNPs that were further characterized: those that resulted in non-synonymous (NS) amino acid changes within ORFs and SNPs located in putative promoter regions. Comparison of these SNPs with genome sequences of three tick transmissible strains was then performed. SNPs that consistently segregated with phenotype were retained. The remaining differences were then targeted sequenced in two additional efficiently transmissible strains. **B**. A total of 9609 SNPs were found between the transmissible St. Maries and the non-transmissible Florida strain (SNPs). This comparison found 4498 non-synonymous SNPs (represented in black), 1630 SNPs found within putative promoter regions (shown in dark grey) and synonymous SNPs (shown in light gray). Whole genome comparison with three transmissible strains allowed removal of 4127 non-synonymous SNPs and 1568 promoter SNPs from further consideration. Finally, Targeted sequencing in additional transmissible strains of 241 non-synonymous and 62 promoter SNPs allowed retention of 35 NS and 14 promoter SNPs as candidate SNPs involved in tick transmission.

**Figure 2:**
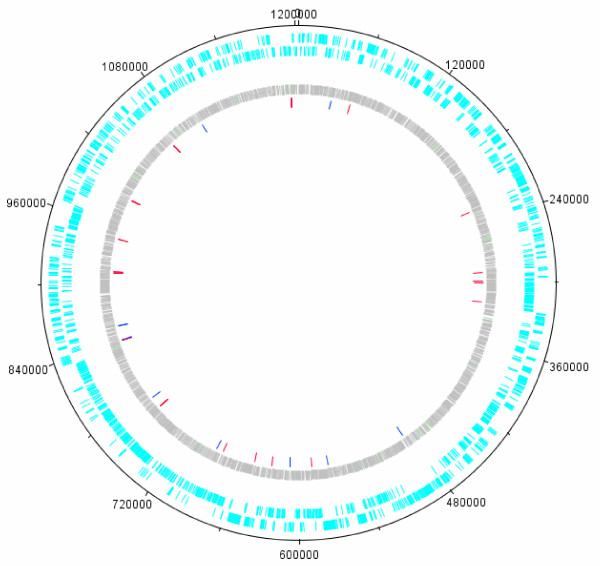
**Location of candidate SNPs on the Florida strain genome.** This circular representation of the Florida genome shows in light blue annotated CDSs; outer circle represents CDSs on the forward strand, inner circle represents the reverse strand, in grey the 9609 SNPs found between the St. Maries and the Florida strain genomes. The elements in light green are miscellaneous features annotated in the genome. In the inner most circle 49 candidate SNPs found through comparative genomics are shown. Red bars show the position of candidate non-synonymous SNPs within CDSs. Dark blue bars show candidate SNPs found within putative promoter regions.

Comparison of the poorly transmissible Florida strain with the efficiently transmitted St. Maries strain produced a total of 9,609 SNPs evenly distributed throughout the genome (Figure 1, Figure 2, and Additional file 1). Two types of SNPs were further characterized: those that resulted in non-synonymous amino acid changes within genes and SNPs located in putative promoter regions. For the purposes of this study, putative promoters were defined as intergenic regions immediately 5^′^ to translation start sites. Global comparison of these SNPs with genome sequences of three efficiently transmitted strains, Puerto Rico, Virginia and South Idaho yielded 241 NS changes within genes, and 62 SNPs distributed in 27 putative promoters. These genes and promoters were then further analyzed in two additional efficiently transmitted strains, 6DE and EMø, by performing targeted sequencing of the regions of interest. The final candidate list included 18 genes that contained at least one SNP encoding a non-synonymous substitution that segregated with transmission status, and 14 SNPs within nine intergenic regions that could potentially affect the transcription of 11 genes (Figure 1, Additional file 1). Altogether, comparative genomics identified 29 candidate genes.

### Transcription analysis of candidate genes

**Figure 3:**
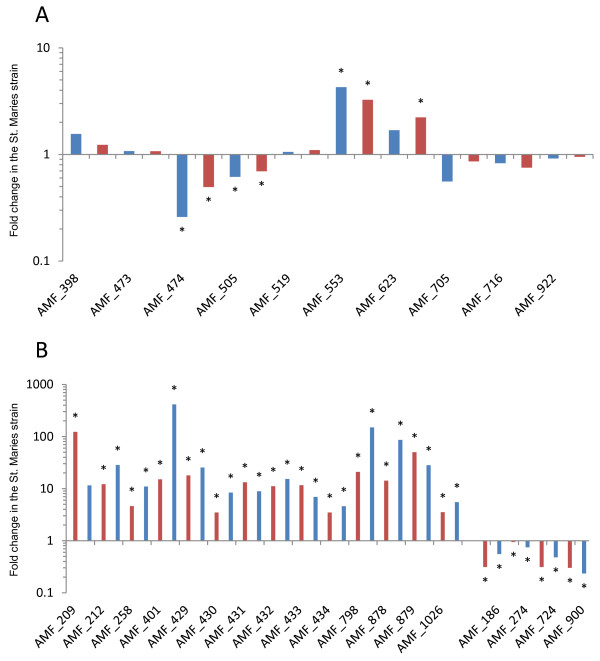
**RNA-seq and qPCR confirm trends in transcriptional changes between strains that differ in their tick transmission status. A**. Fold change in the transmissible St. Maries strain relative to the non-transmissible Florida strain for all promoter candidates expressed in log scale 10. Locus tags for all genes are given on the X axis. Blue bars show the results obtained after evaluating two biological replicates with RT-PCR. Red bars show the fold change obtained using RNA-seq analysis for the promoter candidates across two biological replicates. The asterisk indicates statistical significance at p < 0.05. **B**. Fold change in the transmissible St. Maries strain relative to the non-transmissible florida strain expressed in log_10_. The top 18 differentially transcribed genes identified through RNA-seq across two replicates and two statistical tests and their fold changes are shown. Red bars show results obtained with RNA-seq, blue bars show validation through qPCR. The asterisk indicates statistical significance at p < 0.01.

These 29 genes with SNPs in their coding regions or in their putative promoter regions were analyzed for transcriptional activity by using RT-PCR, which revealed that the 29 candidate genes were transcribed in both the efficiently transmitted St. Maries and the poorly transmissible Florida strain (Additional file 2). For the 11 genes flanking candidate promoter regions, the relative expression ratio was analyzed from two separate infections using *msp5* as a steady state calibrator [22,23]. The fold changes were tested for statistical significance by the pairwise randomization test in two separate infections. Statistical significance of the average fold changes across both biological replicates was tested using an adaptation of the method proposed by Willems et al. [24] (Figure 3A). Four genes were differentially expressed in two biological replicates: AMF_553 showed 4.3 times increased expression in the efficiently transmitted strain (P < 0.05). AMF_474, AMF_505 and AMF_142 showed decreased expression in the highly transmissible strain by ratios of 0.2, 0.6 and 0.7 respectively (P < 0.05). We calculated an expression cutoff by adding 2 standard deviations to the average fold change seen in all the studied genes. Of these differentially expressed candidates, only genes AMF_474 and AMF_553 were below and above the calculated cutoff, respectively. 

### RNA-seq

The transcriptomes of the Florida and St. Maries strains of *A. marginale* were sequenced using three different technologies: 454, Illumina, and Ion Torrent. Roche’s 454 technology provided the longest reads, as expected (Table 1). Interestingly, this technology also yielded the highest percentage of *A. marginale* reads at 37.1%. Although the Illumina platform had the lowest percentage of *A. marginale* reads (4.7% for the Florida strain), this was compensated by depth and was sufficient for quantitative analysis. The use of different platforms allowed us to address some of the challenges of working with obligate intracellular pathogens; as these microbes are dependent on their eukaryotic host cells, RNA samples are significantly contaminated by host transcripts, and RNA preps have been shown to be biased [25]. Our results were corroborated with the different platforms. 

**Table 1:** **Reads mapped to *A. marginale *from three sequencing platforms**

**Platform**	**Strain**	**Total reads**	***A. marginale *reads**	**% of reads mapped to *A. marginale***	**Average matched read length**
**Roche 454**	STM^1^	726,051	269,730	37.1	381.7
	FL^2^	1,018,447	326,440	32.0	396.5
**Ion Torrent**	STM	1,004,747	295,629	29.4	111.0
	FL	2,043,607	577,284	28.2	111.2
**Illumina**	STM	88,650,713	4,604,993	5.2	100
	FL	81,507,967	3,845,853	4.7	100

^1^*STM*: St. Maries strain.

^2^*FL*: Florida strain.

**Figure 4:**
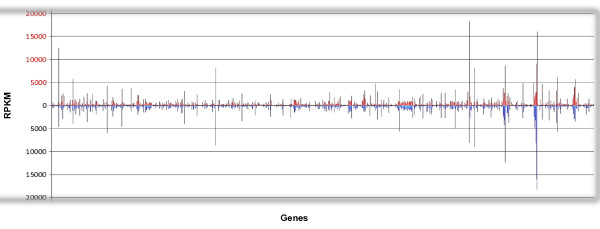
**Newly identified transcriptionally active regions of the genome.** Mapping of cDNA reads to the *A. marginale* genome allowed us to detect regions without previous annotation that exhibited transcriptional activity. **A** shows the region of the St. Maries genome that spans from bp 336042 to 336685. Three different gene identification algorithms did not detect a CDS that would span the length of the transcript. The top panel shows the six reading frames containing forty-five stop codons, shown as black bars. The bottom panel shows some of the mapped cDNA reads in green and red (indicating direction of the read). The grey histogram under the reads represents depth (read height). This transcript was up-regulated in the St. Maries strain by a fold change of 23.7 at p < 1E-10. **B** shows the region of the St. Maries genome that spans from bp 1084944 to 1085520. The newly identified gene is found between genes polA (not shown) and AM1216. One ORF on the leading strand seems to span the length of this transcript and is shown as PUTATIVE_CDS in this figure. This new gene was found to be up-regulated in the transmissible St. Maries strain by a fold change of 6.9 at p < 1E-10.

Transcriptome analysis allowed us to identify putative transcription start sites (TSS) for both strains as a by-product of our study. Seventy putative TSSs were found in the Florida strain and 109 were found for the St. Maries strain (Additional files 3 and 4). The majority of these TSSs are present in both lists, the larger number of high confidence TSSs found in the St. Maries strain can be attributed to the deeper coverage obtained for this strain. Most of the putative 5^′^ untranslated regions (UTR) found were longer than 40 bp in both strains (63.3% in St. Maries and 48.6% in Florida). Fewer putative 5^′^ UTRs were found to be smaller than 40 bp, and the minority were found within the predicted open reading frame (ORF) (Table 2). The 5^′^ UTRs found within annotated CDS suggest that the predictions for these genes were inaccurate and an adjustment in annotation is required. We also identified 70 high confidence operon structures that involved 292 different genes (Additional file 5). Finally, six regions with no previous annotation were found to display high transcriptional activity (Table 3). The six regions showed transcriptional activity in both strains, with two of these newly identified transcripts showing significant differential transcription between the strains. These regions are shown in Table 3 and Figure 4, and are further discussed in the next section.

**Table 2:** **Percentage of putative 5**^′^** UTRs according to length**

**Strain**	**5**^′^** UTRs < 40 bp**	**5**^′^** UTRs ≥ 40 bp**	**5**^′^** UTR within predicted CDS**^**1**^
**St. Maries**	25.68	63.30	11.02
**Florida**	27.15	48.57	24.28

^1^5^′^ UTR within predicted CDS: in this column we list cases where transcript mapping shows that the 5^′^ UTR and TSS are found within the previously predicted and annotated CDS indicating that the previous annotation was incorrect.

**Table 3:** Previously unannotated areas that exhibited high transcriptional activity

**Region**^**1**^	**Length**	**Identity through blastX**	**Gene before**	**Gene after**	**Fold change**
**251313.251855**	543	hypothetical protein AmarV_01231 [Anaplasma marginale str. Virginia]	AM294 pep1	AM259 thiD	0.7
**336042.336685**	644	n/a	AM380	AM382	23.6*
**393765.394740**	976	DNA-binding protein HU [Anaplasma phagocytophilum HZ]	AM434 pdxJ	AM435	1.1
**459343.459783**	441	hypothetical protein AmarM_02282 [Anaplasma marginale str. Mississippi]	AM504	tRNA-Asn-1	1.3
**887245.887579**	335	hypothetical protein PseS9_19739 [Pseudomonas sp. S9]	AM969 bioB	AM973 purL	1.5
**1084944.1085520**	577	hypothetical protein AmarM_05569 [Anaplasma marginale str. Mississippi]	AM1214 polA	AM1216	6.9*

^1^base pair positions spanned by the newly identified regions in the St. Maries genome.

*Statistically significant fold changes are indicated with an asterisk.

### Transcriptome comparison identifies transcriptional differences between strains with contrasting transmission phenotypes

**Figure 5:**
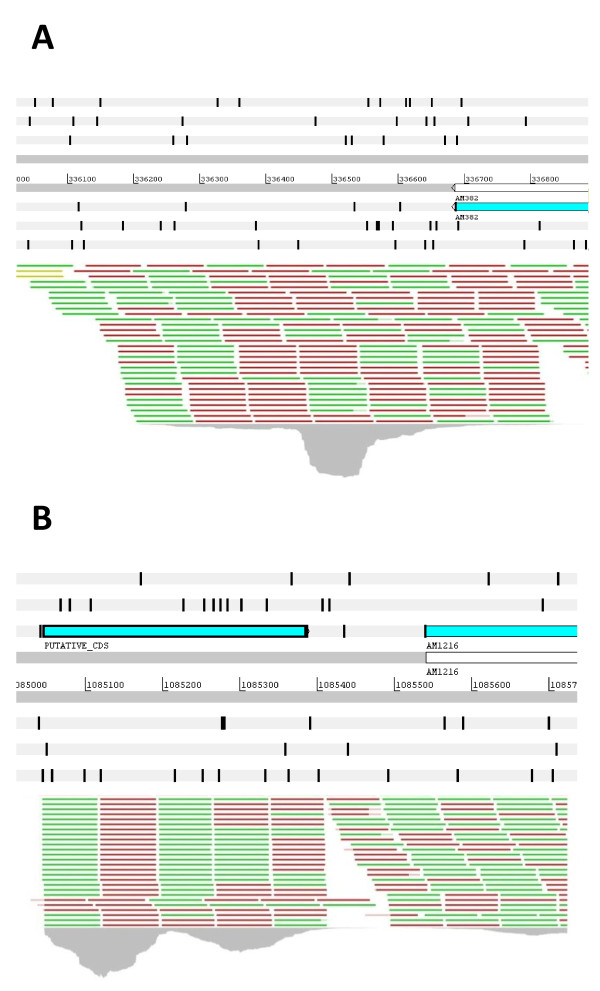
**Whole genome comparison of transcriptional activity in the St. Maries and Florida strains.** The RPKM values for 955 genes found in the Florida strain genome of *A. marginale.* RPKM values are shown on the Y axis. Features are arranged from left to right as they appear in the genome on the X axis. The normalized RPKM values were plotted for each strain. RPKM values for the transmissible St. Maries strain are shown in red in the upper part of the graph; numbers for the non-transmissible Florida strain are shown in light blue in the lower part of the graph. RPKM values for the Florida strain are plotted on the opposite side of the x axis for ease of comparison; they do not represent negative values. Ribosomal RNA (rRNA) genes were subtracted from this comparison.

After normalization, the distributions of the expression values across replicates were compared before evaluating changes in transcription (Additional file 6). Comparing the transcriptomes of the highly transmissible St. Maries strain with the poorly transmissible Florida strain produced a list of 14 genes that are significantly differentially transcribed using our criteria (see Methods) and across replicates (Figure 3B, Figure 5, and Additional file 7). Genes that were found to have a lower transcription level in the poorly transmitted Florida strain are of particular interest considering the examined trait. Significant fold change differences that were constant across replicates ranged from 3.5 to 413.0 (Figure 5, Figure 3B). Of the 10 genes that had significantly low (or absent) transcriptional activity in the Florida strain (Table 4), only one is annotated with a predicted localization: gene AMF_878, coding for outer membrane protein 4 (OMP4). The other nine genes are annotated as hypothetical proteins. Three of these had no mapped reads in any of the different sequencing technologies: AMF_431, AMF_432 and AMF_433. An additional two genes, AMF_429 and AMF_430, which appear to be arranged in an operon with AMF_431-3 (based on reads mapped to the St. Maries genome), are also significantly differentially transcribed (Figure 3B). RNA-seq analysis of the promoter candidates identified by comparative genomics confirmed the RT-PCR results (Figure 3A). Examination of candidates carrying non-synonymous SNPs found significant differential transcription of two genes; AMF_793 and AMF_1026 (shown in Table 4, along with differentially transcribed promoter candidates AMF_474 and AMF_553). These genes have a lower transcription level in the Florida strain by fold changes of 3.5 and 1.5 respectively (p < 1E-10). Finally, two newly identified regions were differentially transcribed. The regions between bp 336042 and 336685 and bp 1084944 and 1085520 in the St. Maries genome (Table 3) were up-regulated by fold changes of 23.6 and 6.9, respectively (p < 1E-10). These regions are shown in detail in Figure 4. In order to determine if these newly identified transcripts could indicate the presence of genes, we searched for ORFs that would overlap these regions. Only two regions: from bp 393765 to 394740 and 1084944 to 1085520 contained ORFs that would span the uninterrupted transcript. Comparisons of replicates were performed in order to account for variation of transcription values within a strain; importantly, the genes that were consistently differentially transcribed across replicates were found to be homogeneously transcribed when strain replicates were compared to each other.

**Table 4:** Candidate genes involved in transmission phenotype segregated by polymorphisms and differential transcription

**Category**	**FL**^**1**^	**St.M**^**1**^	**Product**	**SNPs**	**candidate SNPs**	**SNP location**	**Homologs**^**2**^	**BI**^**3**^
**Differentially transcribed genes**	AMF_433	AM579	Hypothetical protein	1	0	Gene	AP, ER, ECh	TM
	AMF_432	AM579	Hypothetical protein	1	0	Gene	AP, ER, ECh, ECa	-
	AMF_431	AM580	Hypothetical protein	3	0	Gene	AP, ER, ECh, ECa	TM
	AMF_430	AM576	Hypothetical protein	22	0	Gene	AP, ER, ECh, ECa	TM/DS
	AMF_429	AM574	Hypothetical protein	15	0	Gene	AP, ER, ECh	TM
	AMF_798	AM1055	Hypothetical protein	9	0	Gene	AP, ER, ECh, ECa	TM/SP
	AMF_879	AM1165	Hypothetical protein	14	0	Gene	-	TM
	AMF_878	AM1164	Outer membrane protein 4	2	0	Gene	AP	TM/SP
	AMF_401	AM540	Hypothetical protein	12	0	Gene/Prom	-	TM
	AMF_258	AM347	Hypothetical protein	19	0	Gene	AP	TM
**Differentially transcribed genes w/SNPs genescarrying candidate SNPs**	AMF_474	AM632	Ribosome-associated inhibitor A	1	1	Promoter	ER, ECh, ECa	-
	AMF_553	AM748	NADH Dehydrogenase I chain J	1	1	Promoter	AP, ER, ECh, ECa	TM
	AMF_793	AM1048	Hypothetical protein	77	2	Gene/Prom	AP, ER, ECh, ECa	TM/SP
	AMF_1026	AM1352	Hypothetical protein	18	5	Gene/Prom	AP, ER, ECh	TM/DS
**Genes carrying candidate SNPs**	AMF_051	AM071	Hypothetical protein	5	1	Gene	AP, ER, ECh, ECa	TM
	AMF_197	AM265	Hypothetical protein	19	1	Gene	AP, RB	TM
	AMF_264	AM354	Hypothetical protein	21	2	Gene	ER, ECh, ECa	TM/DS
	AMF_265	AM356	Hypothetical protein	65	1	Gene	AP	-
	AMF_269	AM368	Hypothetical protein	43	1	Gene	RB	TM/DS
	AMF_480	AM644	DNA gyrase B	14	1	Gene	AP, ER, ECh, ECa, RB, RC, RR	TM
	AMF_530	AM712	Hypothetical protein	138	1	Gene	-	TM
	AMF_518	AM689	Hypothetical protein	20	1	Gene	AP	-
	AMF_547	AM742	Hypothetical protein	1	1	Gene	AP, ER, ECh, ECa	DS
	AMF_613	AM823	Hypothetical protein	17	4	Gene	AP, ER, ECh, ECa	-
	AMF_703	AM919	Hypothetical protein	1	1	Gene	AP, ER, ECh, ECa, RB, RC, RR	TM
	AMF_762	AM1001	methionyl-tRNA synthetase	23	1	Gene	AP, ER, ECh, ECa, RB, RC, RR	TM/DS
	AMF_764	AM1005	aspartokinase	13	1	Gene	AP, ER, ECh, ECa	TM/DS
	AMF_824	AM1091	D-Ala-D-Ala carboxypeptidase	33	6	Gene	AP, ER, ECh, ECa, RB, RC, RR	TM/SP
	AMF_893	AM1183	lipoprotein-releasing transmembrane protein	8	3	Gene	ER, ECh, ECa	TM/DS
	AMF_1037	AM345	Hypothetical protein	4	1	Gene	-	-

^1^*FL*: Locus tag in the Florida strain *St.M*: locus tag in the St. Maries strain.

^2^*AP*: *Anaplasma phagocytophilum*, *ER*: *Ehrlichia ruminantium*, *ECh*: *Ehrlichia chaffeensis*, *ECa*: *Ehrlichia canis*, *RB*: *Rickettsia bellii*, *RC*: *Rickettsia conorii*, *RR*: *Rickettsia rickettsia.*

^3^*BI*: Bioinformatics *TM*: Transmembrane domain, *SP*: Signal peptide, *Prom*: promoter, *DS*: Deleterious substitution.

### RT-PCR and validation of RNA-seq results

The 18 genes that were the most differentially transcribed across replicates were analyzed by using RT-PCR to confirm the RNA-seq results. Fold change in transcription was evaluated and compared with RNA-seq analysis. As shown in Figure 3B, transcriptional changes were confirmed and statistically significant in all but one of the analyzed genes. Gene AMF_209, found to be more highly transcribed in the St. Maries strain by 124 fold was not consistently up-regulated across both replicates by RT-PCR (up-regulated by 18.2 and 1.3 fold in separate replicates) and, therefore, its fold change was not statistically significant.

### Gene characteristics/bioinformatics

Table 4 shows the 30 genes that were selected as candidates. Genes that were found to be differentially transcribed through RNA-seq and RT-PCR are shown on top of Table 4; genes with candidate SNPs and differential transcription are shown in the middle of Table 4. The rest of the genes contain non-synonymous SNPs that segregated through comparative genomics. The length of the candidate genes varies, with AMF_530 being the longest at 10,479 bp and AMF_1037 the shortest at 240 bp. Twelve of the candidate genes are annotated as hypothetical proteins (Table 4). Genes AMF_474, AMF_553, AMF_480, AMF_762, AMF_764, AMF_824, AMF_893 and AMF_878 are orthologs of genes with known functions. Genes downstream from promoter SNPs included one translation inhibitor (AMF_474) and one gene involved in energy consuming processes, nuoJ (AMF_553). Genes containing non-synonymous substitutions included orthologs for DNA gyrase (AMF_480), a tRNA synthase (AMF_762), an aspartate kinase (AMF_764), a carboxypeptidase involved in cell envelope biogenesis (AMF_824) and a lipoprotein releasing protein (AMF_893). A role in transmission is not immediately apparent for these genes, in fact, it is not surprising that more than half of the candidates were of unknown function due to the lack of information on the determinants of tick transmission. A search for related genes revealed that 18 of the candidate genes had homologs in the tick-transmissible human pathogen *Anaplasma phagocytophilum* (Table 4). Ten genes, AMF_051, AMF_433, AMF_432, AMF_431, AMF_430, AMF_429, AMF_547, AMF_613, AMF_762, AMF_893, AMF_798, and AMF_793 also had homologs in tick transmitted *Ehrlichia* species. Only genes AMF_197, AMF_264, AMF_269, AMF_480, AMF_703, AMF_824 and AMF_893 had homologs in three tick transmitted *Rickettsia* species. Additionally, hypothetical candidates AMF_1037, AMF_879, AMF_401 and AMF_530 had no homologs in the GenBank database. These findings provide two mutually exclusive scenarios: if a gene with homologs in the aforementioned tick-transmitted organisms is responsible for the trait of interest, this suggests a common mechanism within a bacterial order or family. Alternatively, a gene unique to *A. marginale* would favor a species-specific scenario.

Four of the genes encoded transmembrane domains and signal peptides predicted through multiple algorithms: AMF_798, AMF_793, AMF_824 and AMF_878. The results obtained for AMF_878 are not surprising, as it is annotated as outer membrane protein 4 (OMP4). Twenty-three genes had significant scores for transmembrane domain predictions but did not contain signal peptides. Analysis of non-synonymous SNPs using the SIFT algorithm [26] predicted eleven to be deleterious; these substitutions are reported in Additional file 8.

## Discussion

Pairing comparative genomics with high throughput RNA-seq analysis allows for identification of sequence and transcriptional differences on a genome wide scale. In the present study, comparative genomics reduced a list of candidate SNPs from 9,609 to 49 SNPs that segregate with transmission status, including 35 that encode non-synonymous substitutions within 18 genes and 14 residing within nine putative promoters that could affect transcription of 11 genes. Of the putative promoter SNPs, we retained only those that affected the transcription of adjacent genes, leaving just 2 SNPs affecting two genes, reducing the overall list to 37 candidate SNPs affecting 20 genes. Deep sequencing and comparative expression analysis found an additional 10 genes whose transcription between strains with distinct transmission efficiencies is significantly different. Transcriptome analysis also revealed two previously un-annotated regions that were differentially transcribed between the strains of interest. This produced a final list of 30 genes and two newly identified transcriptionally active regions that segregate with tick transmission.

Our combined approach allowed us to map SNPs that segregate among *A. marginale* strains with divergent transmission efficiencies. Such subtle differences have been shown to have dramatic effects on organism biology. A single non-synonymous SNP in the envelope protein gene E1 of the Chikungunya virus is directly responsible for a change in vector specificity that caused an epidemic in the Reunion Island in 2004 [27]. One SNP in the FimH adhesion gene from a commensal strain of *E. coli* modified this strain’s affinity for monomannose receptors, correlating directly with increased uroepithelium affinity and allowing detrimental bladder colonization [21]. Similarly, a SNP within the promoter of the nitrate reductase gene cluster narGHIJ was shown to be responsible for the different nitrate reductase phenotype shown by the almost identical *Mycobacterium bovis* and *Mycobacterium tuberculosis*, bacterial species with identical gene content [28].

Comparative genomics identified 20 genes with at least one SNP that segregated with transmission phenotype. The lack of information on the microbial determinants of tick transmission is consistent with the observation that the majority of the genes containing non-synonymous SNPs are of unknown function. Candidate genes with orthologs in other bacterial species do not appear to have an obvious involvement in the phenotype of interest. Three genes: AMF_798, AMF_793 and AMF_824, were predicted to have both signal peptides and transmembrane domains. The presence of signal peptides and transmembrane domains implies membrane localization of the proteins, and thus, these proteins would be more likely to interact with vector molecules and therefore effect transmission. Out of the 35 non-synonymous candidate SNPs, a little under a third were predicted to be deleterious (Additional file 8). Gene AMF_1026 carries the highest number of deleterious substitutions with a total of three non-synonymous SNPs. This gene was also found to be up-regulated in the efficiently transmitted strain through RT-PCR. Interestingly, it also had a SNP in its promoter region. This promoter SNP did not segregate with the rest of the transmissible strains and therefore was not retained as a candidate. Polymorphisms were retained as candidates if six efficiently transmissible strains consistently diverged with the nucleotide found in the poorly transmitted Florida strain. Candidate SNPs included non-synonymous changes in ORFs and SNPs found in putative promoter regions.

Two genes with candidate SNPs in their putative promoter regions were found to be differentially transcribed. AMF_553, more highly transcribed in the St. Maries strain, is annotated as NADH dehydrogenase I chain J (nuoJ). This is part of the membrane arm of respiratory complex I, a conserved proton pumping NADH:ubiquinone oxidoreductase in bacteria [29]. Another closely associated gene from this complex, nuoL, has been found to be up-regulated in the related organism *Rickettsia conorii* while dealing with osmotic stress [30], suggesting that enhancement of NADH dehydrogenase expression in a vector-transmitted bacterium could be related to an adaptation strategy necessary to survive in the changing osmolarity of a feeding tick [31]. AMF_474, more highly transcribed in the Florida strain, contains conserved domains for a modulation protein, the ribosome associated inhibitor A (RaiA) also known as protein Y (PY). This protein is a cold-shock induced ribosome binding protein that inhibits translation [32]. PY binds exclusively to the 30S subunit of the 70S ribosome, and preventing the formation of initiation complexes by preventing the binding of mRNA and initiator fMet-tRNA to the ribosome [33]. When temperature levels return to 37°C, initiation of protein synthesis overcomes the PY inhibition as tRNA compete more effectively with PY in elevated temperatures. Related bacterial species which are also transmitted by *D. andersoni*, such as *Rickettsia rickettsii*, are known to enter “dormant” stages within ticks [34]. Subsequent reversion of this state, in a process termed “reactivation”, is thought to be due to an increase in temperature when the arthropod feeds on the mammalian host. Therefore these observations suggest an interesting scenario as this gene was up-regulated in the low transmission efficiency Florida strain. The low transmission phenotype could be due to a halt in translation produced by an up regulation of PY during cold shock response.

The three aforementioned differentially transcribed genes were identified through comparative genomics. Although all three carried SNPs in their promoter regions, only two were retained as candidates. This exposes a limitation of the approach that was used in this study: polymorphisms that do not segregate with all the highly transmissible strains may still contribute to the phenotype of interest. In order to confirm the differences in transcription revealed through RT-PCR and to find further changes in transcriptional activity that the strategy might have overlooked; the transcriptome of two strains with contrasting transmission phenotypes were compared. Genome wide comparison of transcriptional activity confirmed our RT-PCR results and found an additional 10 genes that were significantly differentially transcribed. Of these 10 genes only one had a predicted localization: AMF_878 corresponds to OMP4, an outer membrane protein and member of the pfam 01617 superfamily [11]. Among the remaining genes with no functional annotation three genes stood out as they exhibited a complete lack of transcriptional activity in the poorly transmitted Florida strain: AMF_431, AMF_432 and AMF_433. These genes appear to be arranged in an operon along with AMF_429 and AMF_430, according to the tiling of reads mapped in the St. Maries strain. AMF_429 and AMF_430 were also significantly differentially transcribed between the strains. Genes AMF_429, AMF_431 and AMF_433 contain high scoring conserved domains for tail and head/tail connector phage proteins, with the highest similarity found to phage proteins from *Wolbachia spp*., a related bacterial symbiont of arthropods. Although this could open interesting possibilities, as phages play an important role for *Wolbachia spp*. within the arthropod host [35], no mobile elements or intact prophages have been identified in *A. marginale*[11].

Typically, pathogenic bacteria that cycle between arthropod and mammalian hosts modify their transcriptional profiles to adapt to these different environments [36]. One of the major difficulties involved in examining gene regulation of obligate intracellular pathogens is the low amounts of bacterial RNA, which is co-isolated with large amounts of host RNA. In order to overcome the limited amount of bacterial RNA, previous transcriptomic studies interrogating genes used for obligate intracellular survival were conducted using mimetic conditions of infection in an *in vitro* environment [37-39]. While these studies provide insight into a limited number of genes regulated by specific cues, they are not representative of natural infection. Exposing the related pathogen *R. rickettsii* to different environmental conditions that mimic its transition from arthropod to mammalian host showed a surprisingly minimal transcriptional response, with less than 10 genes changing more than 3-fold in expression level [37]. This could indicate that pathogens in the order *Rickettsiales* do not regulate genes specifically for growth within mammalian or tick cells but contain a conserved set of genes that are required for growth in both environments. The obligate intracellular habitat of pathogens in this Order may offer such a stable environment that the necessity for gene regulation is much less than that of facultative intracellular pathogens. Our study searched for transcriptional differences between strains with contrasting transmission profiles in the natural host of our model organism.

The use of different sequencing platforms in this study was instrumental in confirming significant and consistent changes in transcriptional activity. It has been shown that different RNA preparation and selection procedures in deep sequencing experiments can lead to measurable over- or under-representation of particular RNAs [25]. This study proved that utilizing different technologies allowed for control of sources of potential bias in RNA sequencing: all three platforms used for our study gave the same results. Making use of various platforms was also instrumental in our goal of describing the *A. marginale* transcriptome with the highest possible accuracy. In bacteria, the overwhelmingly high numbers of reads in combination with relatively small genome sizes has led to the assumption that complete or nearly complete transcriptomes are being analyzed. However, selecting for prokaryotic sequences in an ocean of eukaryotic RNAs makes accurate representation of RNA populations daunting. Few attempts have been made at describing the transcriptome of obligate intracellular pathogens through RNA-seq; notably, to date, this has been done for *Chlamydia* species [38,39] and the tick-transmitted pathogen *A. phagocytophilum*[40]. The deepest analysis generated 854,242 reads that mapped to the 1.23 Mb *Chlamydia pneumonia* genome [39]; we mapped up to 2,990,921 reads per replicate to *A. marginale*’s 1.2 Mb genome. To enrich for prokaryotic sequences, previous attempts at characterizing obligate intracellular microbial transcriptomes used differential centrifugation of *in vitro* grown bacteria in order to separate the bacteria from host cells. This procedure is likely to stress the bacteria and skew their transcriptional profile. Enrichment for our samples was performed by selective hybridization once RNA populations were collected. Although Mastronunzio et al. used a similar enrichment procedure; they only detected 187,284 reads, representing 11% of the CDSs in the *A. phagocytophilum* genome [40]. In this study, 99% of the CDSs in the *A. marginale* were detected through transcriptional analysis.

Analyzing transcriptional profiles with RNA-seq allows us to evaluate “snapshots” in time of bacterial transcriptomes; therefore, it is essential to generate data from more than one replicate to provide a broader more reliable picture of transcriptional changes. The depth and reproducibility of this RNA-seq data set allowed for mapping of the physical structure of the *A. marginale* transcriptome; including previously unreported transcriptionally active regions and 5^′^ UTR length. Six regions with no previous annotation were detected in both strains; two of these were differentially transcribed. The role of these transcripts is uncertain as only two of these were predicted to contain ORFs. The majority of the high confidence 5^′^ UTRs were longer than 40 bp in both strains. Previous studies of TSSs have shown that only a very small portion of 5^′^ UTRs are longer than 40 bp in bacteria [41,42]. As 5^′^ UTRs have been involved in regulation processes in bacteria, further investigation of these elements might reveal translational and transcriptional roles [43]. Additionally, mapping of transcriptional data allowed us to define 70 putative operon structures that involved 292 genes, showing that at least 30% of the genes are polycistronic. Although RNA-seq allows us to study polycistronic messages on a genome wide scale, the depth of this technique coupled with tiling arrays have shown that the concept of simple operons is questionable. Differential expression of consecutive genes within operons and condition dependent modulation highlight the complexity of transcriptional regulation in bacteria [44].

## Conclusions

This study takes advantage of the high interstrain diversity of this intracellular bacterium to significantly reduce the number of candidate differences that could be involved in the tick transmission phenotype. Marrying next generation sequencing approaches allowed us to generate a list of genes differing at the transcriptional and sequence levels in strains with contrasting transmission status. Transformation of the transmission deficient allele into a transmission competent strain will facilitate functional analysis of these genes in order to determine their role in transmission by the arthropod host. Although the successful transformation of *A. marginale* has been achieved [45,46], stable targeted gene replacement has not been accomplished and is a necessary next step for determining the role of these genes in tick transmission. Identification of genes involved in tick transmission in our model will provide an important first step toward the development of novel control strategies for tick-borne pathogens, such as transmission-blocking vaccines.

## Methods

### Ethics statement

Animal experiments were approved by the Institutional Animal Care and Use Committee at University of Idaho, USA, in accordance with institutional guidelines based on the U.S. National Institutes of Health (NIH) Guide for the Care and Use of Laboratory Animals.

### Strains

The Florida, St. Maries, Virginia, Puerto Rico, South Idaho, EMΦ and 6DE strains used in this study have been described in detail elsewhere [47-51]. The St. Maries, Virginia, Puerto Rico, South Idaho, EMΦ and 6DE strains are reproducibly transmitted by the Reynolds Creek stock of *D. andersoni*[13,14,16,17,52,53]. The Florida strain has not been successfully transmitted by any tick species, including the Reynolds Creek stock [15,18,19].

### Comparative genomics

The accession numbers for the strains used are: St. Maries: CP000030.1, Florida: CP001079.1, Viriginia: ABOR00000000.1, Puerto Rico: ABOQ00000000.1, South Idaho: AFMY00000000.1. MUMmer v3.1 [54] was used to compare as previously described [8] to compare the Florida and St. Maries strains. SNPs encoding synonymous substitutions were not further analyzed. The runMapping program of the Newbler suite v2.5.3 (454 Life Sciences) was used with default settings to compare all reads from the Virginia, Puerto Rico, and South Idaho strains to the completed Florida and St. Maries genomes. All remaining SNPs from the initial comparison were then checked against the three strains; if the Florida sequence was matched in any of the highly transmissible strains, that SNP was removed as a candidate. Illumina sequencing of the St. Maries strain was used to evaluate the frequencies of the SNPs found between the Florida and St. Maries strain. SNPs that were found at 100% frequencies were highlighted in Additional file 1.

### Targeted sequencing

The remaining SNPs were examined via targeted sequencing of the South Idaho, EMΦ and 6DE strains [50,55]. Primers were designed by aligning the SNP-containing region from the Florida and St. Maries strains and selecting primers to flank the polymorphism. The resulting amplicons were generated from genomic DNA, cloned into pCR4-TOPO (Invitrogen) and sequenced in both directions using BigDye v3.1 chemistry on an ABI 3130XL (Applied Biosystems). Sequence analysis eliminated candidates as described above. All candidate SNPs were resequenced in the Florida strain, to verify the original genomic sequence.

### Comparative transcriptional analysis

Total RNA was isolated from *A. marginale*-infected blood using TRIzol (Invitrogen), per manufacturer directions. Expression was measured using quantitative reverse transcription PCR using the SYBR Green ER RT-PCR Kit (Invitrogen). Briefly, 1 ug of RNA was processed with the Superscript III First strand kit (Invitrogen) to obtain cDNA. Copy numbers were corrected to more closely reflect transcript levels based on reverse transcription efficiency [52]. The steady state, single copy gene *msp*5 was used to calibrate the RT-PCR. Relative expression ratios were calculated by a mathematical model, which includes efficiency correction of individual transcripts through the REST software [56]. This software uses the Pair Wise Fixed Reallocation Randomization Test to assess the statistical significance of the RT-PCR results when comparing the relative expression of the promoter candidates in both the Florida and St. Maries strains. A differential expression fold cutoff value of 3.2 was established by calculating the mean of the average ratios observed for all genes analyzed in this study plus 2 standard deviations. In order to assess the statistical relevance of the findings across two biological replicates, an adaptation of the standardization method proposed by Willems and coworkers was used [24]; this includes three basic steps: log transformation, mean centering and autoscalling. After standardizing the data, statistical significance of the fold changes observed between the strains across both experiments was determined by calculation of 95% confidence intervals. This procedure was applied to each candidate gene and was also used for verification of transcriptional differences found by RNA-seq.

### RNA-seq

The accession number for this RNA-seq study is: SRP014580. Two Holstein calves negative for *A. marginale* by MSP5 cELISA, C1322 and C1323, were inoculated with the Florida and the St. Maries strains, respectively. Infection levels were tracked by analysis of Giemsa-stained blood smears to calculate the percentage of parasitized erythrocytes (PPE). Blood samples were taken at similar levels of parasitemia (3.5 and 4% PPE). Total RNA was isolated from *A. marginale*-infected blood using TRIzol (Invitrogen) per the manufacturer’s directions. Eukaryotic sequences were negatively selected through hybridization using the MICROBEnrich kit (Ambion). For samples processed for 454 and Ion Torrent technologies, probes for bacterial ribosomal RNAs from the Ribominus kit (Invitrogen) were added during the subtractive hybridization procedure. For samples processed for Illumina, the Duplex‐Specific thermostable nuclease (DSN) normalization protocol was applied. Data was processed using CLC Genomics Workbench (CLC Bio). Mapping parameters were adjusted to map a maximum number of reads to the reference bacterial genomes. The distribution of the expression values for all samples was analyzed and compared. Normalization by quantiles was applied to adjust the distributions for further comparison. Fold changes with respect to RPKM (Reads Per Kilobase per Million mapped reads) values were calculated [57]. Two different tests were applied to evaluate the statistical significance of fold changes: Kal’s and Baggerly’s statistical tests on proportions [58,59]. Comparisons of replicates were performed in order to account for variation within a strain. These comparisons showed very little variation: a maximum of 2% of genes had fold changes above or below 1. As variation within strains was assessed we proceeded to compare the differentially transmitted strains. In order to establish transcription fold change cutoffs, the relationship between the p-values of the statistical tests applied and the magnitude of the difference in expression values of the samples was plotted and evaluated. This was done in order to arrange genes along dimensions of biological and statistical significance [60]. Genes whose log2 fold change was above and below 2 and -2, respectively, and whose -log10 p-value was above 10 in both replicate comparisons and under both statistical tests were selected for further evaluation (Additional file 7).

Areas of the genome that were not previously annotated and showed >0.5 coverage (average sequence data coverage depth) were reported when reads were unambiguously mapped to the *A. marginale* genome [42].

The relative performances noted in Table 1 for the different sequencing technologies should not be directly compared, as this study was not designed to compare these platforms. As has been noted [25], different library preparations and sequencing technologies favor recovery of different transcripts. The goal of using multiple technologies was to verify that under- or over-represented transcripts in any strain were not being favored by the technology used.

### Putative start site identification

Putative transcript start sites were identified using the rules proposed by Passalacqua et al. [42]: briefly; genes with continuous coverage extending into a codirectional upstream gene were identified as members of an operon. If the signal “dropped off” in the intergenic sequence upstream of the open reading frame, we designated the point at which coverage dropped to 0 as the putative transcriptional start site. Coverage depth was calculated for every position of each genome, and all genes considered had an average coverage score >0.5 above the calculated average coverage signal. Putative TSSs that were found with the highest confidence (i.e. TSSs present in all replicates) were grouped in two different tables according to the length of the 5^′^ UTRs, less or more than 40 bp.

### Bioinformatic analysis of candidates

In order to rank the candidates, two different criteria were established. The first, termed “biological plausibility of association”, examines the annotation of the currently available genomes and the predicted function of the candidate gene, using existing knowledge about biology and the studied phenotype [61]. In other words, is the candidate gene likely to be involved in the examined phenotype according to its known or predicted function? The second criterion involves the use of three *in silico* analyses. The presence of signal peptides in the candidate genes was assessed by using SignalP 4.0 [62]. Transmembrane domains were predicted using two distinct algorithms: TMpred and Dense Alignment Surface (DAS) methods [63]; only genes with transmembrane domains predicted by both algorithms were reported. The “Sorting Tolerant From Intolerant” (SIFT) algorithm [26] uses a sequence homology-based approach to classify amino acid substitutions, and was used to predict if substitutions in the candidate alleles detrimental or tolerated by the protein. The search for ORFs in newly identified transcriptionally active regions was performed using three different tools: CLC Genomics Workbench (CLC Bio), NIH’s ORF finder ( http://www.ncbi.nlm.nih.gov/gorf/gorf.html) and ORF ( http://bioinformatics.biol.rug.nl/websoftware/orf/orf_start.php).

## Abbreviations

CDS: Coding DNA Sequence; NS: Non-Synonymous; ORF: Open Reading Frame; RPKM: Reads Per Kilobase per Million mapped reads; SNP: Single-Nucleotide Polymorphism; UTR: UnTrasnlated Region.

## Competing interests

The authors declare that they have no competing interests.

## Authors’ contributions

SAP, MJD, GHP, KAB conceived the experiments; SAP, MJD, DD performed the experiments; SAP, MJD, DD, GHP, KAB analyzed the data; SAP, MJD, GHP KAB wrote and edited the manuscript. All authors read and approved the final manuscript.

## Supplementary Material

Additional file 1 **Nucleotide polymorphisms between the St. Maries and Florida strains.** SNPs between the St. Maries and Florida strains are listed here together with the nucleotides reported for all the additional reported nucleotides. Click here for file

Additional file 2 **Absolute expression values of candidate genes.** Gene identifications are provided on the x axis and the copy number per ml of blood on the y axis. The black bars represent the numbers obtained for the Florida strain and the white bars the numbers for the St. Maries strain. Transcription of all candidate genes is shown together with the calibrator MSP5. Click here for file

Additional file 3 **Mapping of putative TSS and 5**^′^** UTRs length in the St. Maries strain.** The location and length of 5^′^ UTRs in the St. Maries strain are reported. Click here for file

Additional file 4 **Mapping of putative TSS and 5**^′^** UTRs length in the Florida strain.** The location and length of 5^′^ UTRs in the Florida strain are reported. Click here for file

Additional file 5 **Operon strucutres found through transcriptome sequencing in the St. Maries strain.** Genes involved in the different operon structures are reported. Click here for file

Additional file 6 **Distribution of the normalized expression values of all replicates analyzed in this study with RNA-seq.** The distribution of the normalized RPKM values for all replicates is plotted in a box plot. RNA-SeqFL1 and RNA-SeqFL2 designate distributions for Florida strain replicates 1 and 2 respectively. RNA-Seq STM 1 and RNA-Seq STM 2 designate RPKM distributions for St. Maries replicates 1 and 2 respectively. The distributions allow for comparisons. Click here for file

Additional file 7 ***A. marginale* genes arranged along dimensions of biological and statistical significance.** A volcano plot shows the relationship between the p-values of a statistical test and the magnitude of the difference in expression values. On the y axis the negative log10 p-values are plotted. On the x-axis the log 2 values of the fold changes seen in whole transcriptome comparison. The red lines highlight the cutoffs for genes that were analyzed further. Only genes populating the upper right and left quadrants of the plot under two different statistical tests (Kal’s and Baggerly’s) were chosen. This plot shows results obtained for Kal’s test. Click here for file

Additional file 8 **Candidate non-synonymous changes predicted to be deleterious by the SIFT algorithm.** Non-synonymous changes predicted to be deleterious by the SIFT algorithm found between the St. Maries and Florida strains are reported. Click here for file
