# Supplementary material for: Comparative genomics and transcriptomics of trait-gene association
Source: BMC Genomics. 2012 Nov 26;13:669. doi: 10.1186/1471-2164-13-669 (PMC3542260; doi:10.1186/1471-2164-13-669)
Supplement: Additional file 2 — Absolute expression values of candidate genes. Gene identifications are provided on the x axis and the copy number per ml of blood on the y axis. The black bars represent the numbers obtained for the Florida strain and the white bars the numbers for the St. Maries strain. Transcription of all candidate genes is shown together with the calibrator MSP5. [file 1471-2164-13-669-S2.pptx]

## Slide 1
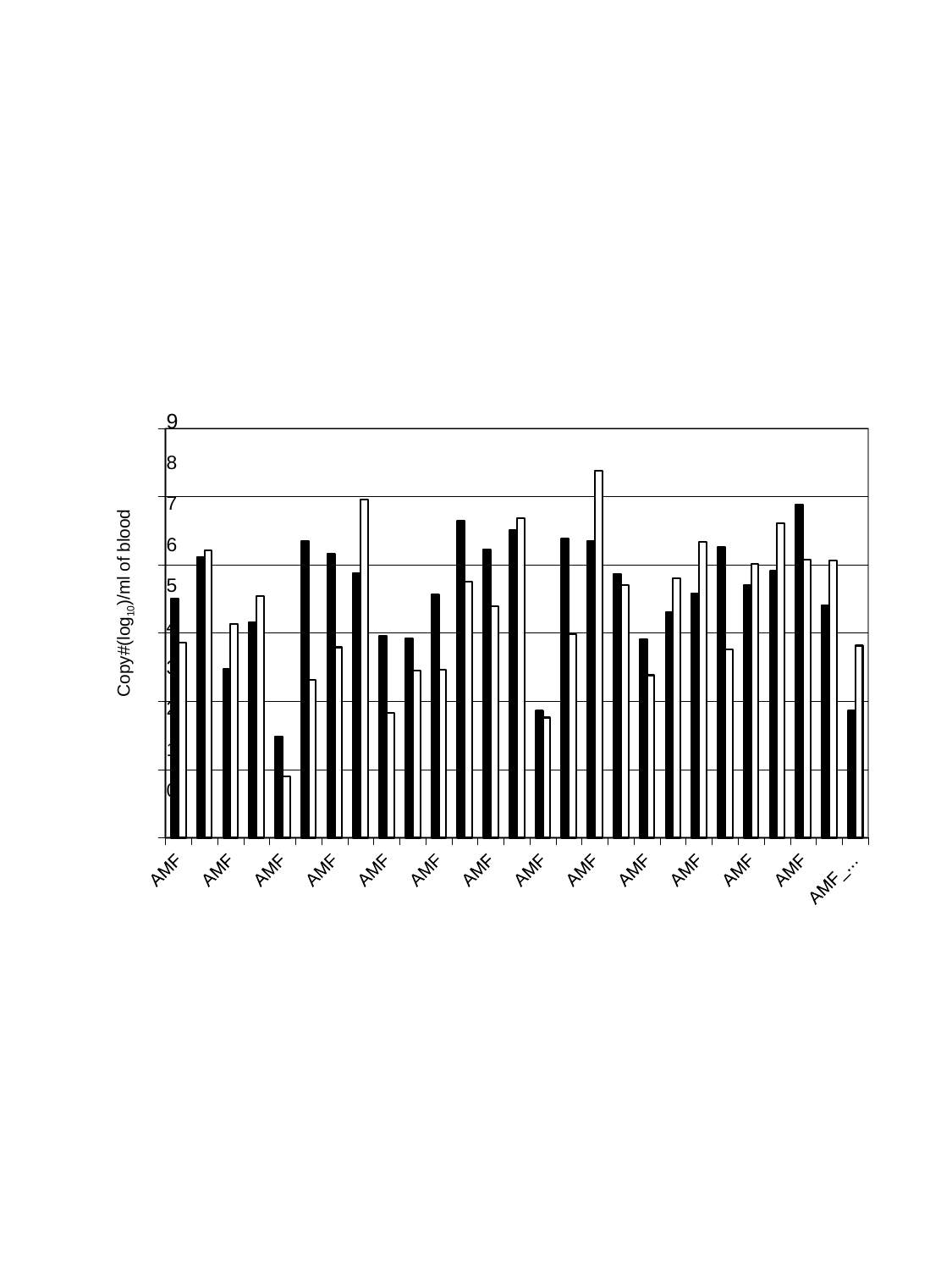

### Chart
| Category | Florida | St.Maries |
|---|---|---|
| AMF_171 | 3230000.0 | 724000.0 |
| AMF_051 | 13000000.0 | 16200000.0 |
| AMF_197 | 297000.0 | 1360000.0 |
| AMF_265 | 1450000.0 | 3520000.0 |
| AMF_398 | 30300.0 | 7990.0 |
| AMF_473 | 22500000.0 | 204000.0 |
| AMF_474 | 14400000.0 | 618000.0 |
| AMF_480 | 7600000.0 | 90000000.0 |
| AMF_505 | 904000.0 | 67400.0 |
| AMF_518 | 845000.0 | 285000.0 |
| AMF_519 | 3710000.0 | 292000.0 |
| AMF_530 | 44700000.0 | 5710000.0 |
| AMF_547 | 16600000.0 | 2440000.0 |
| AMF_553 | 32100000.0 | 48100000.0 |
| AMF_613 | 72500.0 | 57700.0 |
| AMF_623 | 24600000.0 | 951000.0 |
| AMF_703 | 22000000.0 | 237000000.0 |
| AMF_705 | 7330000.0 | 5090000.0 |
| AMF_716 | 817000.0 | 241000.0 |
| AMF_762 | 2030000.0 | 6340000.0 |
| AMF_764 | 3790000.0 | 21900000.0 |
| AMF_793 | 18200000.0 | 576000.0 |
| AMF_824 | 4990000.0 | 10400000.0 |
| AMF_893 | 8100000.0 | 40700000.0 |
| AMF_922 | 76300000.0 | 11800000.0 |
| AMF_1026 | 2520000.0 | 11600000.0 |
| AMF_1037 | 72400.0 | 654000.0 |Copy#(log10)/ml of blood
