# Supplementary figures and images for: Comparative genomics and transcriptomics of trait-gene association
Source: BMC Genomics. 2012 Nov 26;13:669. doi: 10.1186/1471-2164-13-669 (PMC3542260; doi:10.1186/1471-2164-13-669)

## Slide 1
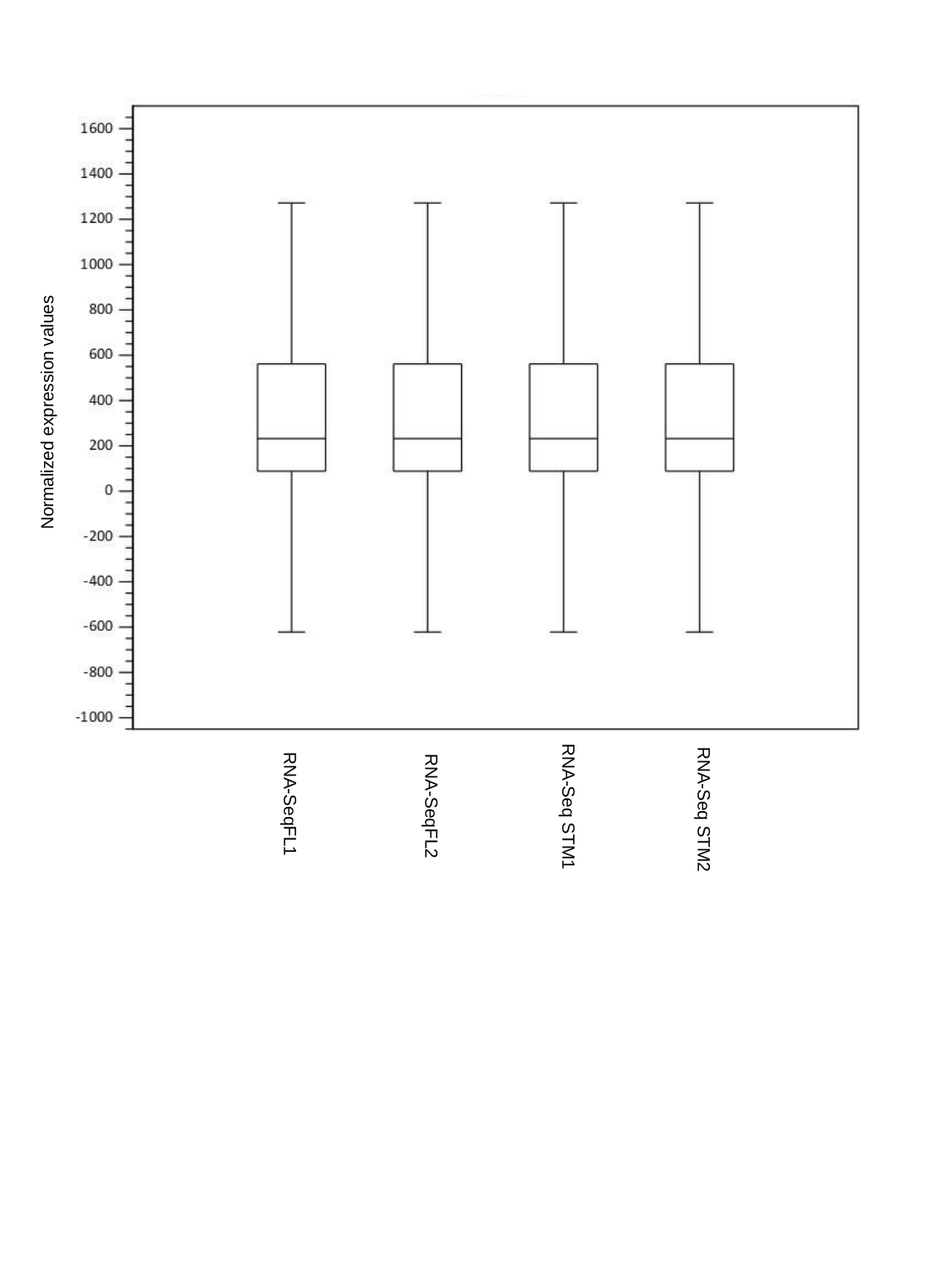

Normalized expression values
RNA-SeqFL1
RNA-Seq STM1
RNA-SeqFL2
RNA-Seq STM2

Supplement: Additional file 6 — Distribution of the normalized expression values of all replicates analyzed in this study with RNA-seq. The distribution of the normalized RPKM values for all replicates is plotted in a box plot. RNA-SeqFL1 and RNA-SeqFL2 designate distributions for Florida strain replicates 1 and 2 respectively. RNA-Seq STM 1 and RNA-Seq STM 2 designate RPKM distributions for St. Maries replicates 1 and 2 respectively. The distributions allow for comparisons. [file 1471-2164-13-669-S6.pptx]

## Slide 1
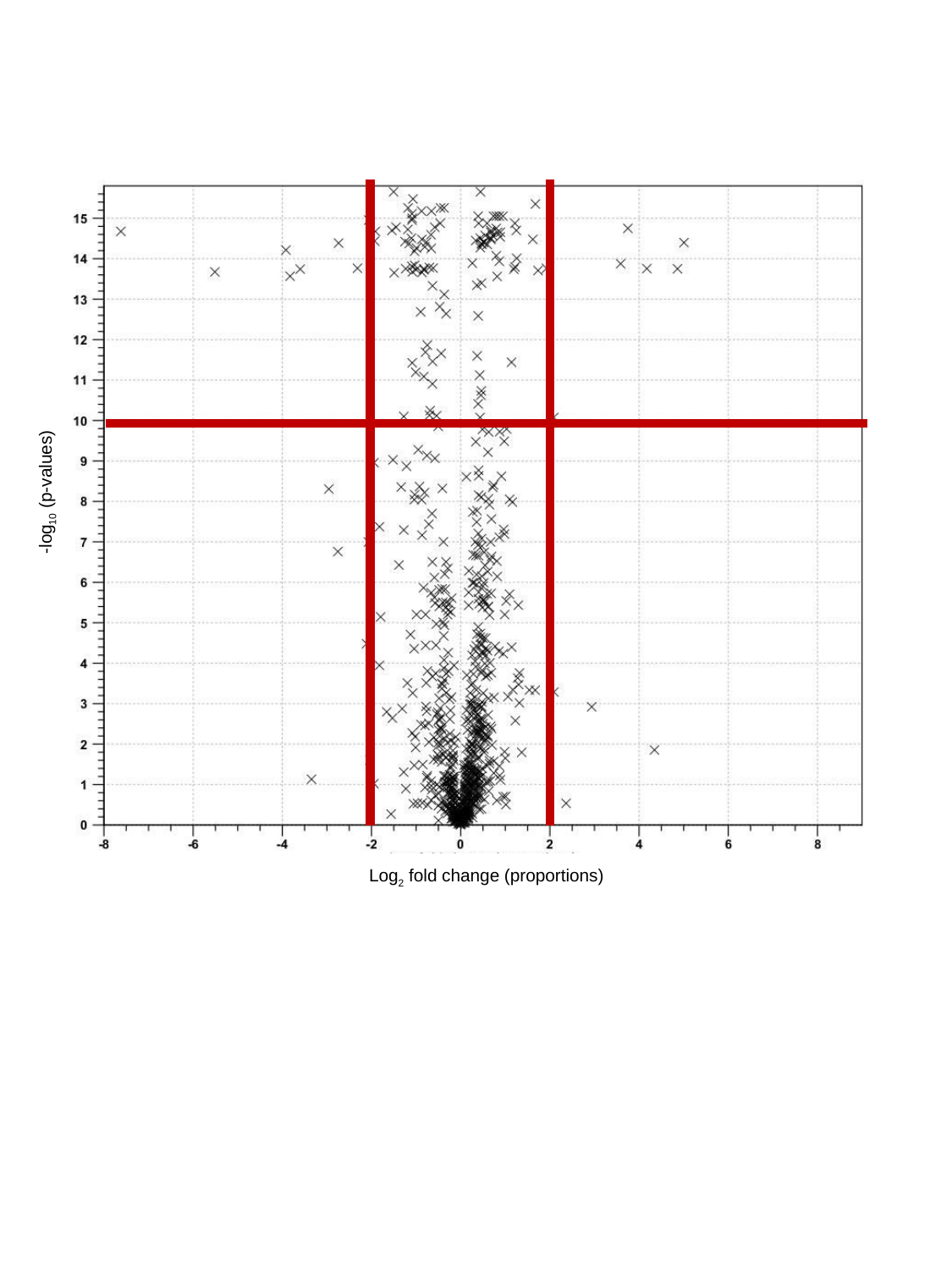

-log10 (p-values)
Log2 fold change (proportions)

Supplement: Additional file 7 — A. marginale genes arranged along dimensions of biological and statistical significance. A volcano plot shows the relationship between the p-values of a statistical test and the magnitude of the difference in expression values. On the y axis the negative log10 p-values are plotted. On the x-axis the log 2 values of the fold changes seen in whole transcriptome comparison. The red lines highlight the cutoffs for genes that were analyzed further. Only genes populating the upper right and left quadrants of the plot under two different statistical tests (Kal’s and Baggerly’s) were chosen. This plot shows results obtained for Kal’s test. [file 1471-2164-13-669-S7.pptx]
