# Supplementary material for: Comparative genomics and transcriptomics of trait-gene association
Source: BMC Genomics. 2012 Nov 26;13:669. doi: 10.1186/1471-2164-13-669 (PMC3542260; doi:10.1186/1471-2164-13-669)
Supplement: Additional file 8 — Candidate non-synonymous changes predicted to be deleterious by the SIFT algorithm. Non-synonymous changes predicted to be deleterious by the SIFT algorithm found between the St. Maries and Florida strains are reported. [file 1471-2164-13-669-S8.docx]

**Supplementary Table S5: Candidate non-synonymous changes predicted to be deleterious by the SIFT algorithm**

| Gene | Description | Position in the Florida genome | Amino Acid in Florida | Amino acid in St. Maries |
| --- | --- | --- | --- | --- |
| AMF_264 | hypothetical protein | 299040 | Glutamine | Arginine |
| AMF_264 | hypothetical protein | 299131 | Serine | Glycine |
| AMF_269 | hypothetical protein | 321307 | Aspartic acid | Cysteine |
| AMF_430 | hypothetical protein | 528313 | Glycine | Aspartic acid |
| AMF_547 | hypothetical protein | 680650 | Cysteine | Tyrosine |
| AMF_762 | methionyl-tRNA synthetase | 911553 | Serine | Arginine |
| AMF_764 | aspartate kinase | 913078 | Methionine | Leucine |
| AMF_893 | lipoprotein-releasing system transmembrane protein | 1061636 | Histidine | Tyrosine |
| AMF_1026 | hypothetical protein | 1195613 | Alanine | Valine |
| AMF_1026 | hypothetical protein | 1195626 | Alanine | Threonine |
| AMF_1026 | hypothetical protein | 1195634 | Valine | Alanine |
